# Studying the Characteristics of Chaos and Fractals of Construction Rocks under Different Loading Velocities

**DOI:** 10.3390/ma15227890

**Published:** 2022-11-08

**Authors:** Nan Wu, Jiyang Fu, Chao Xiong

**Affiliations:** 1 Reserch Center for Wind Engineering and Engineering Vibration, Guangzhou University, Guangzhou 510006, China; 2 School of Physics and Electronic Science, Qiannan Normal University for Nationalities, Duyun City 558000, China

**Keywords:** construction rock, chaotic, fractal, rock damage, logistic map

## Abstract

**Highlights:**

- Rock materials exhibit obvious strain rate effects at different loading velocities.
- The damage evolution process of rock materials contains chaotic features.
- Logical mapping is used to analyze rock material damage–strain information sequences.
- The fractal dimension of rock sample crack is positively correlated with load velocity.

**Abstract:**

Rock is a widely used construction material; its mechanical properties change due to the influence of different load speed. In this study, the split Hopkinson pressure bar (SHPB) was used to test the dynamic properties of rock samples by loading four different pressures (0.05, 0.08, 0.14, and 0.23 MPa). The peak stress of the sample increases from 82.19 to 284.16 MPa, and the particle size of the sample debris decreases from 46.57 to 18.34 mm as the impact pressure increases from 0.05 to 0.23 MPa. As a chaos method in nonlinear dynamics, it is introduced into the quantitative evaluation of the sample at four loading pressures, which is then calculated. The damage evolution process of the sample under four loading pressures is calculated, and the chaotic characteristics contained in the process are analyzed. Based on the logistic mapping, the increase in the load velocity can delay the entry of the damage variable into the period-doubling bifurcation and chaotic states. Finally, the fractal dimension of the rock crack at the corresponding time under different load speeds is calculated, and the results showed that the increase in the load velocity can increase the uniformity of the crack distribution.

## 1. Introduction

Construction rock, as a kind of widely distributed natural geological material, has been widely used in engineering construction [1,2]. Rock materials often contain defects due to long-term geological effects, which show significant ambiguity, nonlinearity and anisotropy on the macroscopic scale [3,4,5,6]. It is an effective method to study the variation characteristics of rock materials on dynamic loading by using chaos theory in nonlinear dynamics.

As a typical form of motion of nonlinear systems, chaotic behavior is characterized by sensitivity to initial values and has very complex fractal and self-similar structures [7,8]. Chaos discrimination based on the Lyapunov index is used to observe information sequences of rock materials and is considered to be an effective method to analyze the failure properties of construction rocks [9]. As a typical representative, Liu [10], based on the Logistics equation, observed the damage evolution sequence in the process of rock loading failure and analyzed the chaotic characteristics of the rock material deformation and failure processes under static loading conditions, and obtained good results. Meanwhile, in fractal theory, Sun et al. [11] provided quantitative guidance for analyzing the stability of a rock mass during excavation engineering by fractal characterization, and Ning et al. [12] studied the fractal characteristics of fatigue failure of coal rock samples. Both studies successfully analyzed the characteristics of rock failure.

Construction rock not only accounts for the action of static load, but it also bears the effect of a wide range of dynamic loads [13,14]. However, the rock material will exhibit significant strain rate effects on dynamic loading, and the properties of the rock will also be significantly different from those on the static loading [15,16,17,18,19,20]. Sunita et al. [18] studied the dynamic loading properties of three kinds of rocks (compact basalt, metadolerite and granite) by SHPB, electron microscope and X-ray equipment. It was concluded that the dynamic peak strength increased by 4.14–7.52 times in the range of the test strain rate. Meanwhile, Millon et al. [21] filled the gap of information that exists regarding dynamic behavior of sedimentary rocks under strain rates between 10^0^ and 5.2 × 10^2^ s^−1^. Gong et al. [20] analyzed the mechanical properties and failure behavior of rock-like materials in the range of 2.7 to 4.0 × 10^5^ MPa/s, where an increase in peak strength and elastic modulus of rock together with a decrease in peak strain and dynamic failure duration in the post-peak period are shown.

Xia et al. [22] described the development and research progress of dynamic compression, dynamic Brazil test and dynamic notched semi-circular bend (three rock dynamic testing methods recommended by the International Society for Rock Mechanics) and summarized the different properties of rock materials under dynamic loading. Zhang et al. [23] summarized the research work in this field, and some popular semi-empirical rate-dependent equations for the enhancement of dynamic mechanical properties were given. The above research work explored the properties of the rock materials on dynamic loading conditions. It is very meaningful research work, and the theory of chaotic fractals can provide an effective exploration path for this research work.

In this paper, the split Hopkinson pressure bar (SHPB) test equipment is used to analyze the characteristics of rock materials at different loading speeds. The evolution law of chaos contained in the damage–strain relation is analyzed. At the same time, the variation of dimension damage of rock samples at different loading speeds is studied by fractal theory.

## 2. SHPB Test

### 2.1. Sample Preparation

**Figure 1:**
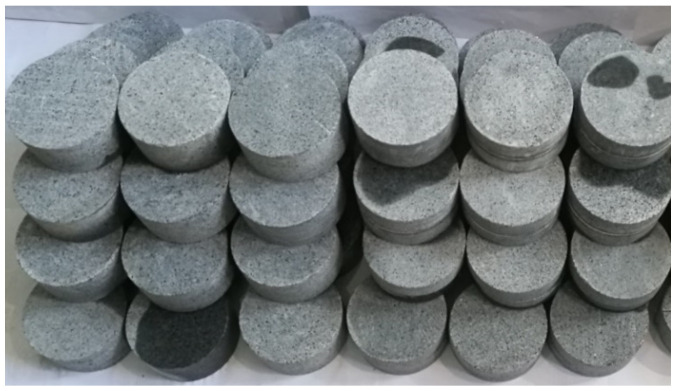
The rock samples.

Construction rock samples were drilled from Xia’men mining area, China. The samples were Φ80 × 40 mm cylinders that exhibited scratches smaller than 0.025 mm with less than 0.025 mm deviation in the flatness. Meanwhile, the samples were cleaned and dried in a natural environment, as shown in Figure 1. These samples were tested in four series (DT-A/B/C/D), depending on the test conditions.

### 2.2. Experimental Setup and Test Method

**Figure 2:**
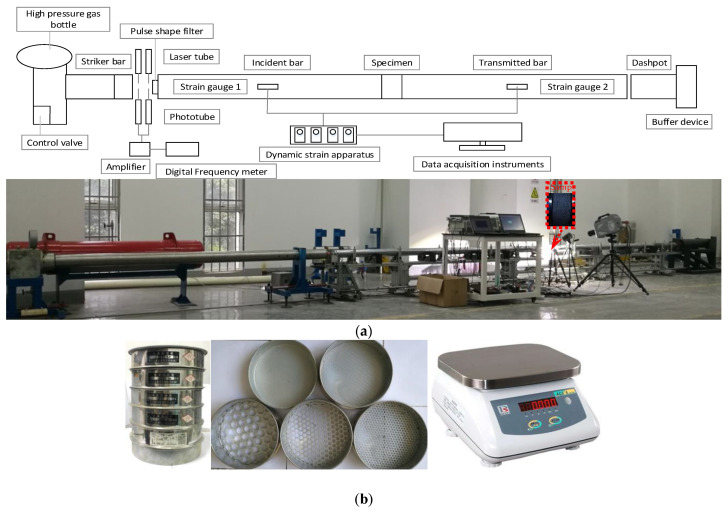
Test equipment. (**a**) SHPB apparatus system. (**b**) Screening device.

The split Hopkinson pressure bar (SHPB) test device with a diameter of 100 mm was used in this study, as shown in Figure 2 (bullet length: 800 mm, incident rod length: 4100 mm, transmission rod length: 3100 mm). Butyl rubber wafers (diameter: 30 mm, thickness: 1.5 mm) were used as waveform shapers. The purpose of waveform shaping is to make the waveform approximate to the sinusoidal curve shape and to lengthen the rising time of the waveform, so as to help the sample achieve stress balance as soon as possible during loading. In the long-term use of the equipment (SHPB), this specification waveform shaper was found to meet the test requirements very well.

Before each test, the impact load is carried out to determine the interference degree of noise to the circuit (strain gauge and Wheatstone bridge), and to remove high frequency noise by adjusting the low-pass filter in the dynamic strain gauge to ensure credibility of the experimental data.

In this test, four different loading pressures (0.05, 0.08, 0.14, and 0.23 MPa) were set up to produce different stress wave amplitudes. The samples were tested at each impact speed, and multiple tests were performed under each operating condition to omit outliers and to ensure good test repeatability. A high-speed camera system was used to record the destruction process of the samples.

The screening tool used in the test is shown in Figure 2b. The apertures were 2.0, 5.0, 10.0, and 20 mm, and the sample was sieved into 0.0–2.0, 2.0–5.0, 5.0–10.0, 10.0–20.0, and 20.0–80.0 mm (80 mm is the diameter before the sample is broken) groups of particle size. The five groups of particles were numbered according to particle size from small to large (I = 1, 2, 3, 4, 5). The weighing instrument was a high-precision electronic scale (Model: ACS-S) with a range of 1 kg and an accuracy of 0.1 g.

## 3. Results

### 3.1. Stress–Strain Curves

The stress–strain curve is an important expression of information reflecting the deformation and failure process of rock under external load. It is of great significance to accurately understand the physical and mechanical properties of rock materials and to guide engineering practice.

**Figure 3:**
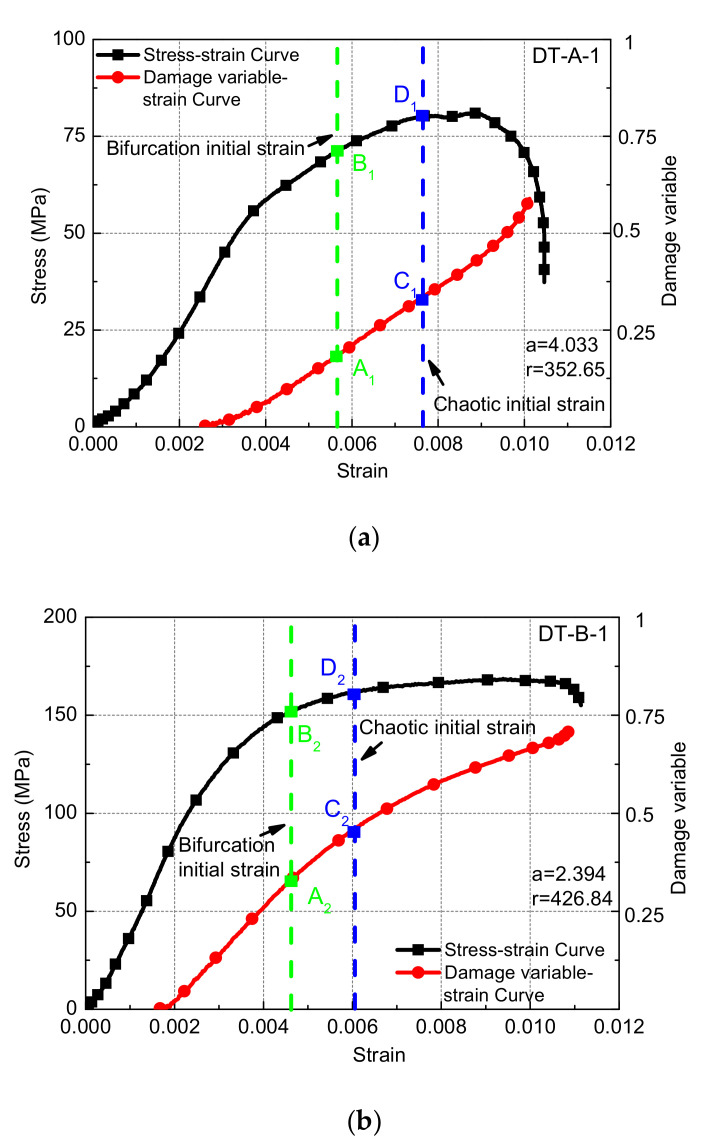

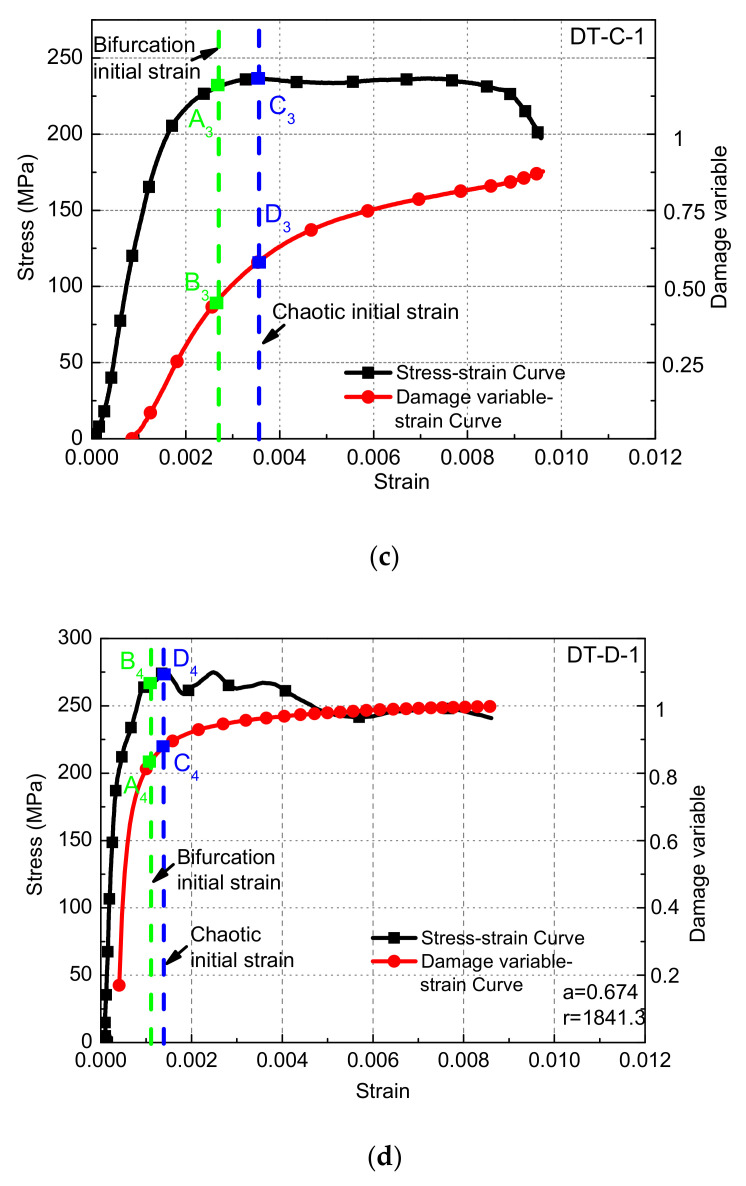
The stress/damage–strain relationship curves of samples at different loading conditions: (**a**) 0.05 MPa; (**b**) 0.08 MPa; (**c**) 0.14 MPa; (**d**) 0.23 MPa.

**Figure 4:**
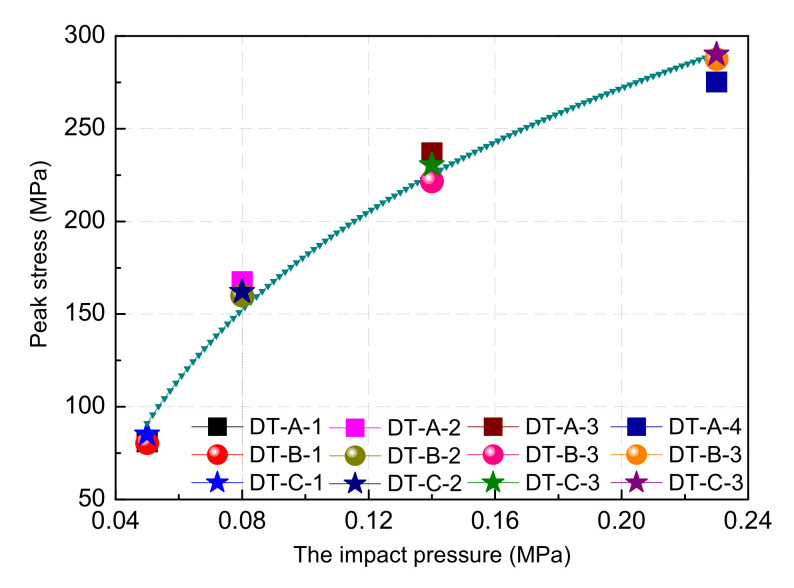
The relationship between peak stress and impact pressure.

The rock sample exhibited a significant strain rate effect with the increase in load velocity, as shown in Figure 3 and Figure 4 and Table 1. With the impact pressure increases from 0.05 to 0.08, 0.14, and 0.23 MPa, the peak stress (mean) increases from 82.19 to 163.18, 229.62, and 284.16 MPa, respectively, increases of 98.54%, 179.38%, 245.74%, and the process shows the law of power function ($y=30300.56x^{0.00473}+29816.3$), This is because the sample is input with more energy per unit time at high-speed loading conditions, and the energy dissipation rate of rock materials is higher than that at low-speed loading conditions, such that the crack along the weakest path at a low strain rate evolved along the maximum energy dissipation path at a high strain rate. The cracks in the rocks continued to change from intergranular to transgranular, and the peak intensity of the sample on the macro scale increased, showing a significant strain rate effect [24,25,26].

### 3.2. The Sample Debris after Loading

After the sample is damaged by the impact load, the relationship between the number and the size of blocks with the loading speed is one of the basic problems of rock dynamics. The fracture state of rock samples is different at different loading speeds, and the fragmentation degree of broken pieces often has certain mass distribution characteristics. In order to characterize this relationship, the average block size is used as a measure:

(1) $$\bar{x}=\frac{\sum x_{i}\eta_{i}}{\sum \eta_{i}}$$

where $\bar{x}$ is the average size of the broken sample (mm); $x_{i}$ is the average size of debris remaining on each stage of the screen (mm); $\eta_{i}$ is the percentage of the mass of the debris when the average size is $x_{i}$, %.

**Figure 5:**
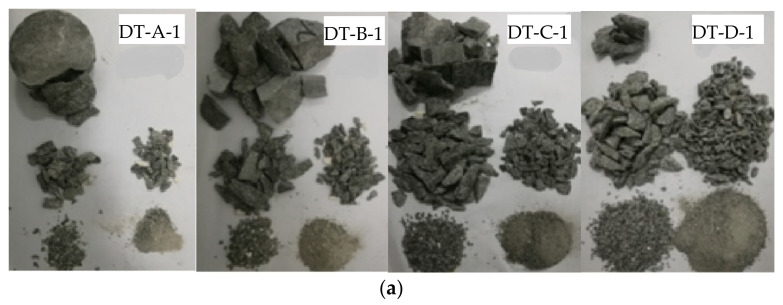

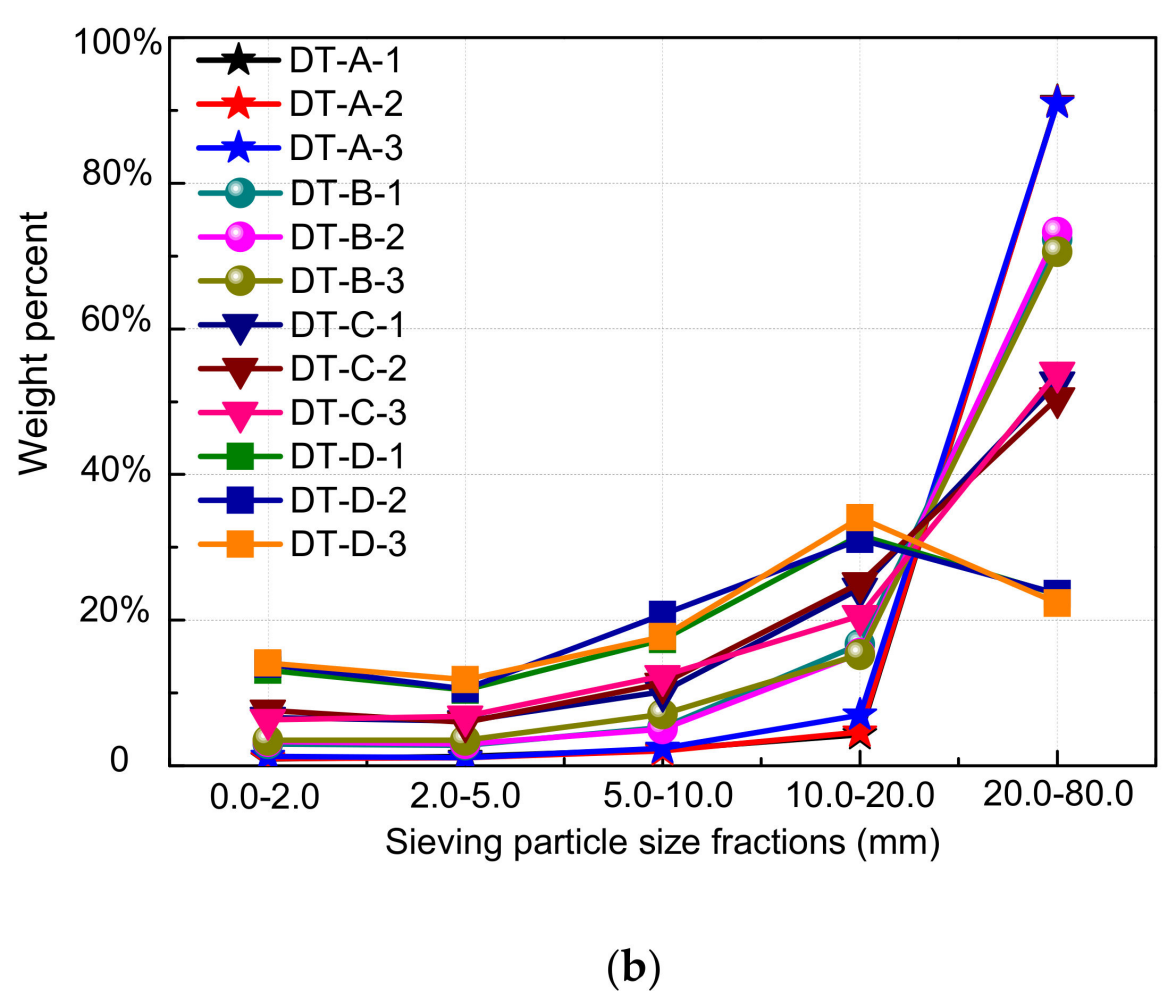
The sample debris after loading: (**a**) photo of the typical distribution of the sample debris; (**b**) figure of variation of the particle size.

The broken rock samples are collected one by one, sieved and weighed one by one. The typical distribution of the sample debris after screening and separation at different impact speeds is shown in Figure 5a. The percentage of the mass fraction of the debris is gradually changed with the increase in the impact pressure (the average particle diameter is 46.57 mm at 0.05 MPa; 38.96 mm at 0.08 MPa; 30.83 mm at 0.14 MPa; and 18.34 mm at 0.23 MPa). The variation of the particle size of the rock sample is shown in Figure 5b and Table 2.

As the loading speed increases, the average particle size of the rock debris decreases gradually, and the degree of fracture is more severe. However, the cracks are basically carried out parallel to the loading direction, and the broken rock debris mostly exhibits a prism shape without significant change. The number of small-sized rocks as debris increases sharply when the loading speed increases, mainly because the cracks are more densely developed and the friction between the prisms is more severe.

## 4. Discussion

### 4.1. The Chaotic Characteristics of Rock Sample Damage Evolution

#### 4.1.1. Rock Damage

Liu [10] obtained the rock damage evolution law based on the Lemaitre strain equivalent hypothesis [26] and Weibull distribution (Equation (2)).

(2) $$D=\frac{k}{1+e^{a-r\varepsilon }},\;0<k\leq 1$$

In Equation (2), D is the damage variable ($D=1-\frac{A^{\prime }}{A}$, A is the initial cross-sectional area of the non-destructive material, $A^{\prime }$ is the effective bearing area (net area) after the material is damaged); ε is the strain; a is the parameter reflecting the degree of initial damage, r is the internal growth rate of damage evolution, and k is the maximum damage degree.

**Figure 6:**
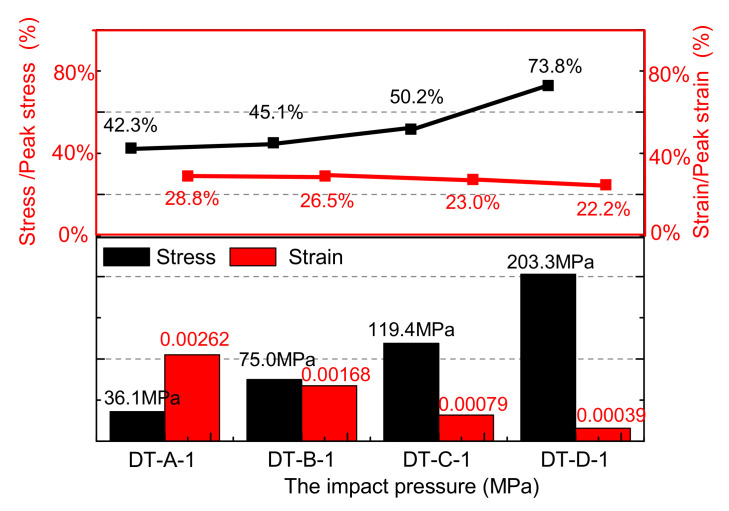
The effects of different loading speeds on stress–strain of samples.

Typical rock sample stress–strain and damage–strain curves at different loading speeds are shown in Figure 3a–d. As the impact pressure increases from 0.05 to 0.08, 0.14, and 0.23 MPa, the stress value of the damage initiation time of the sample increases from 36.1 to 75.0, 119.4, and 203.3 MPa, and the ratio of the value to the corresponding peak intensity increases from 42.3% to 45.1%, 50.2%, and 73.8%, respectively. On the contrary, the corresponding strain at this time was reduced from 0.00262 to 0.00168, 0.00079, and 0.00039, respectively. The higher the loading speed, the shorter the destruction process of the sample. The damage value of the sample is increased from 0.589 to 0.715, 0.972, and 0.996, as shown in Figure 6.

This is because the sample inputs more energy per unit time as the impact velocity increases, the crack that is along the weakest path at the low speed load is converted along the maximum energy-consuming path, a large number of tiny cracks are not fully expanded, and macroscopic cracks caused by transgranular fracture are formed. The appearance of macroscopic cracks causes the material to be in a state of failure. Therefore, the strain corresponding to the peak stress moment of the sample will be smaller at high-speed loading. Meanwhile, the phenomenon of the damage–strain curve of the sample gradually changes from a concave curve into a convex curve, due to the damage surge phase changes from one peak to the peak before.

#### 4.1.2. Chaotic Characteristics

As a heterogeneous natural material at the mesoscale, the dynamic response of rock under high-speed impact is a nonlinear behavior. Based on the analysis method of chaos theory, the assumption that rock materials are isotropic is the premise for subsequent analyses. The deformation and failure process of rock at the external loading can be obtained from the sequence information of the experiment, so as to study the characteristics of the rock damage. Deriving the damage evolution Equation (2), the differential form of the damage evolution equation can be obtained [10]:

(3) $$\frac{dD}{d\varepsilon }=rD(1-\frac{D}{k})$$

The above equation can be written as the following iterative expression:

(4) $$D_{n+1}^{\prime }=\mu D_{n}^{\prime }(1-D_{n}^{\prime })=f(D_{n}^{\prime }),\;n=0,1,2,3,\cdots \;\mu =r\Delta \varepsilon +1,\;D_{n}^{\prime }=\frac{r\Delta \varepsilon }{(r\Delta \varepsilon +1)k}D_{n}$$

**Figure 7:**
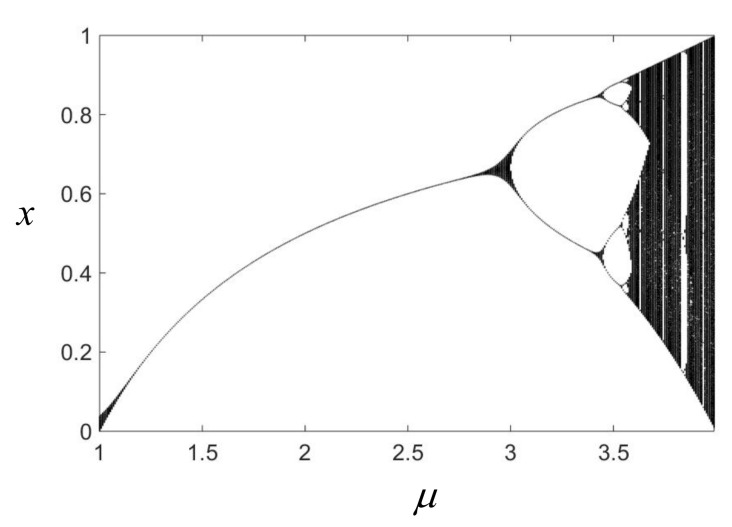
Solution graph of logistic equation.

The mapping f is a logistic mapping reflecting the evolution of the generalized damage variable $D_{n}$. Since the function $f(D_{n}^{\prime })$ is nonlinear, and the inverse map $D_{n}^{\prime }=f^{\prime }(D_{n+1}^{\prime })$ cannot be defined singly, such nonlinear maps are irreversible [27]. For the control parameters μ∈[0,4], $x_{n+1}=f(x_{n})\in [0,1]$ is the invariant interval of the logistic map. In this interval, as μ increases, the logistic map undergoes a period-doubling bifurcation and finally enters a chaotic state, as shown in Figure 7.

As from Figure 3, based on the experimental data, the period-doubling bifurcation points A_1~4_(μ∈[3,3.5699)) of the generalized damage variable at different loading speeds are obtained, and the stress value B_1~4_ corresponding to point A_1~4_ is also obtained. At the same time, the starting point C_1~4_(μ∈[3.5699,4)) and the corresponding stress values D_1~4_ of the generalized damage variable entering the chaotic state are obtained.

**Figure 8:**
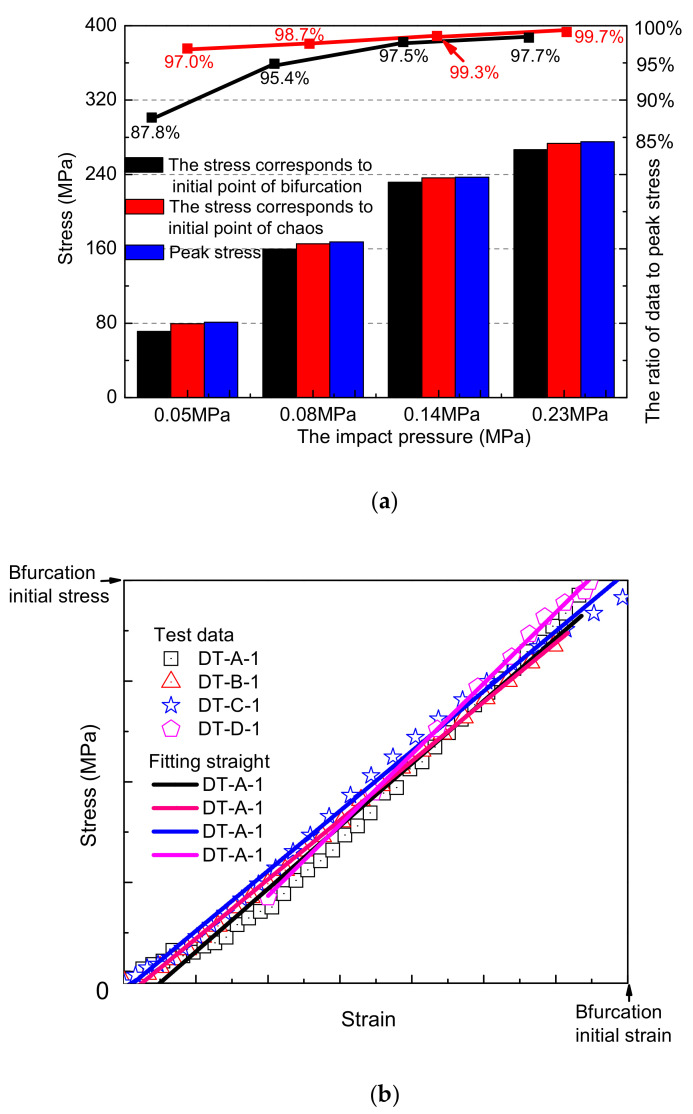
The chaotic characteristics of rock damage evolution: (**a**) influence of loading speed on chaotic characteristics of samples; (**b**) similarity of damage evolution.

From Figure 8a, the stress value corresponding to the generalized damage variable at the period-doubling bifurcation points to the stress peak increases with the increase in the loading speed; when the impact pressure is 0.05 MPa, the ratio is 87.8%. As the impact pressure is increased to 0.08, 0.14, and 0.23 MPa, respectively, the value becomes 95.4%, 97.5%, and 97.7% (Figure 8a, black fold line). The ratio of the stress value to the peak stress corresponding to the chaotic starting point of the generalized damage variable has a similar law (Figure 8a red line). Increases in the impact velocity can delay the generalized damage variable enter the periods of period-doubling bifurcation and chaotic states.

### 4.2. Effect of Loading Velocity on Chaotic Characteristics

The damage–strain cures of the sample from the initial damage time to the start of the period-doubling bifurcation at different loading speeds were treated as dimensionless; the data were fitted separately, as shown in Figure 8b. It can be found that the difference in the loading speed has little influence on the curve form of the stage. The damage–strain curve shows linear growth, and the evolution law (trend) shows similarity. The difference in the fitted linear slope is less than 7%.

This is because the voids inside the rock material are tightly closed at this stage. Usually only a small amount of plastic deformation occurs, which is the initial stage of the damage process. The increase in the load speed only affects the duration of the process and does not affect the damage mode, and the generalized damage variable attracts a fixed point $D^{*}=\frac{\mu -1}{\mu }$($\underset{n\to +\infty }{\lim}f^{n}(D_{0})=\frac{\mu -1}{\mu }$) in the logistic equation. Due to the strain rate effect of the rock material, the difference in duration makes the stress of the sample different at the end of the stage.

### 4.3. The Fractal Characteristics of Rock Sample Damage Evolution 

#### 4.3.1. The Damage Fractal Dimension

The damage material is (equivalent to) the porous media, and the damage evolution process is equivalent to the micropore evolution process in the material. The fractal dimension of the rock crack reflects the characteristics of the crack and the complexity of the section, which directly affect the judgment of the properties of the rock materials. The Hausdorff dimension (Equation (5)) and the box dimension method are used to calculate the damage situation corresponding to the period-doubling bifurcation and the chaotic states while using the fractal geometry covering method to quantitatively investigate the statistical self-similarity of the rock crack [27,28,29,30,31].

(5) $$D_{H}=\underset{\delta \to 0}{\lim}\frac{\ln N(\delta )}{\ln(1/\delta )}$$

where δ is the metric indicating the measure; N(δ) represents the number of targets covered by the scale; $D_{H}$ is the covered dimension

**Figure 9:**
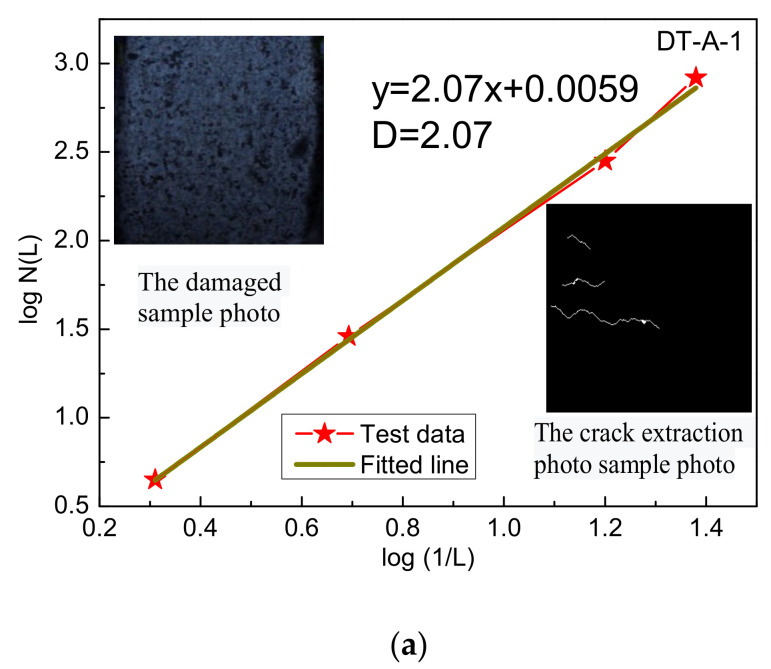

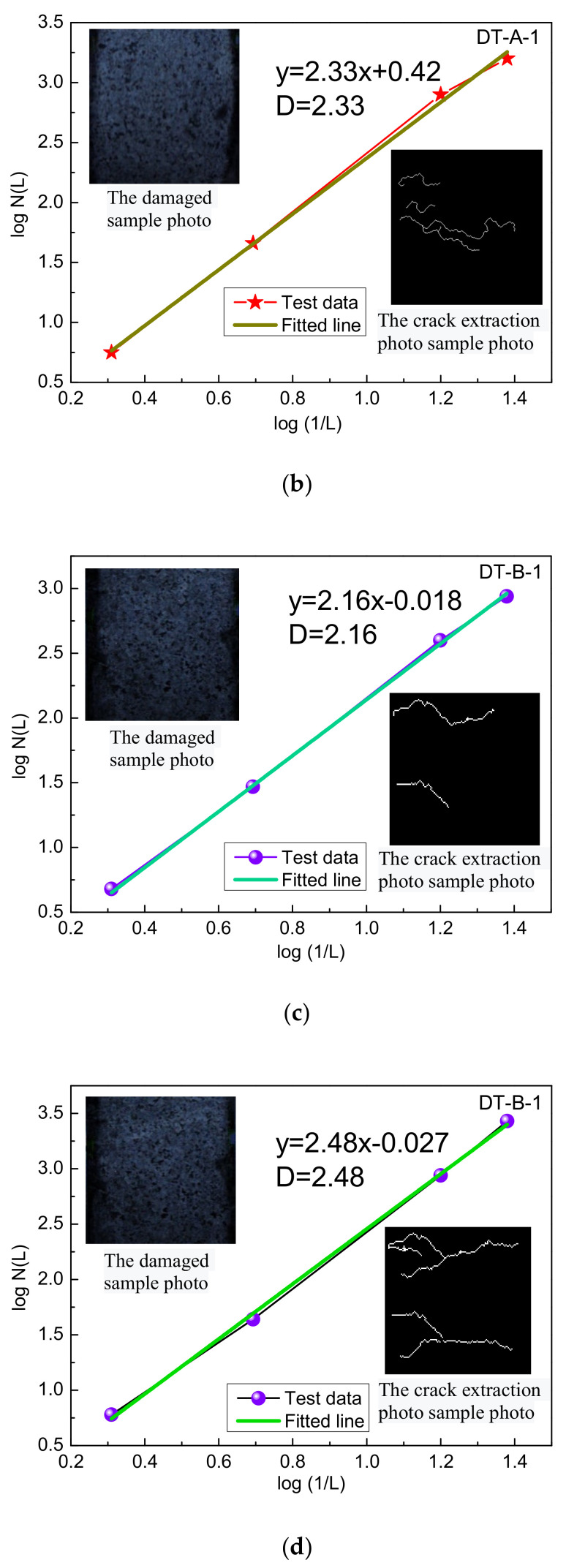

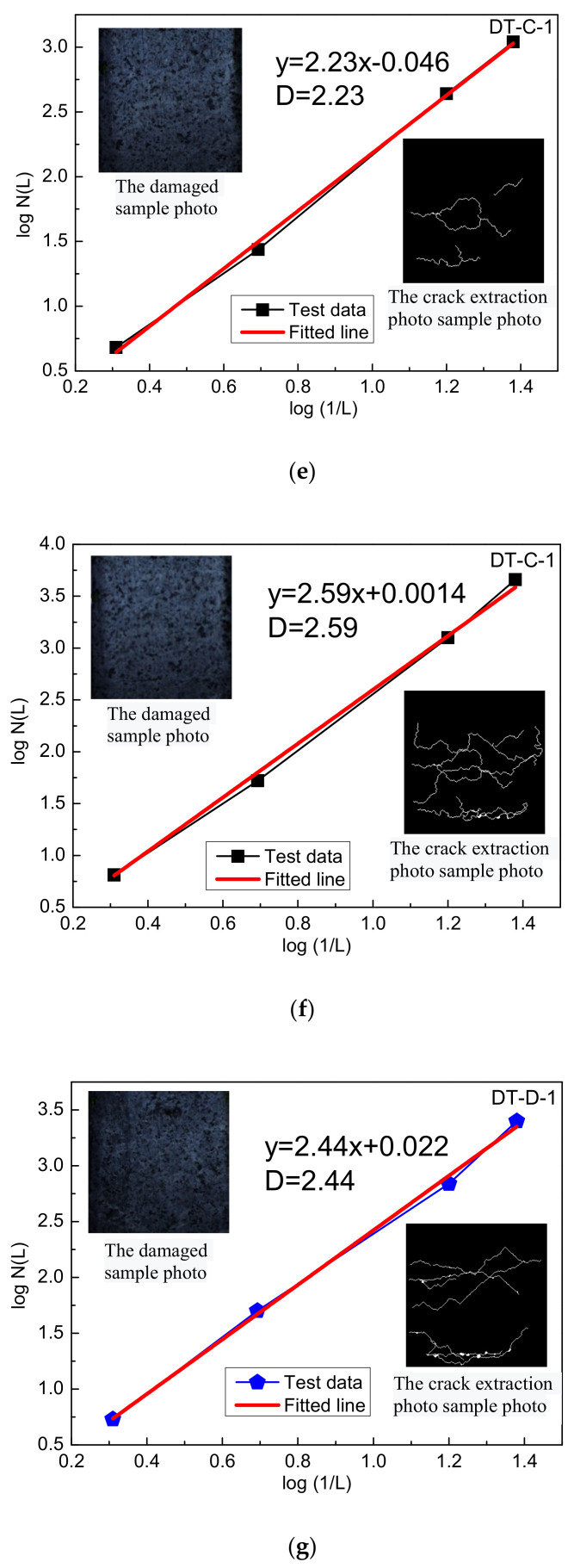

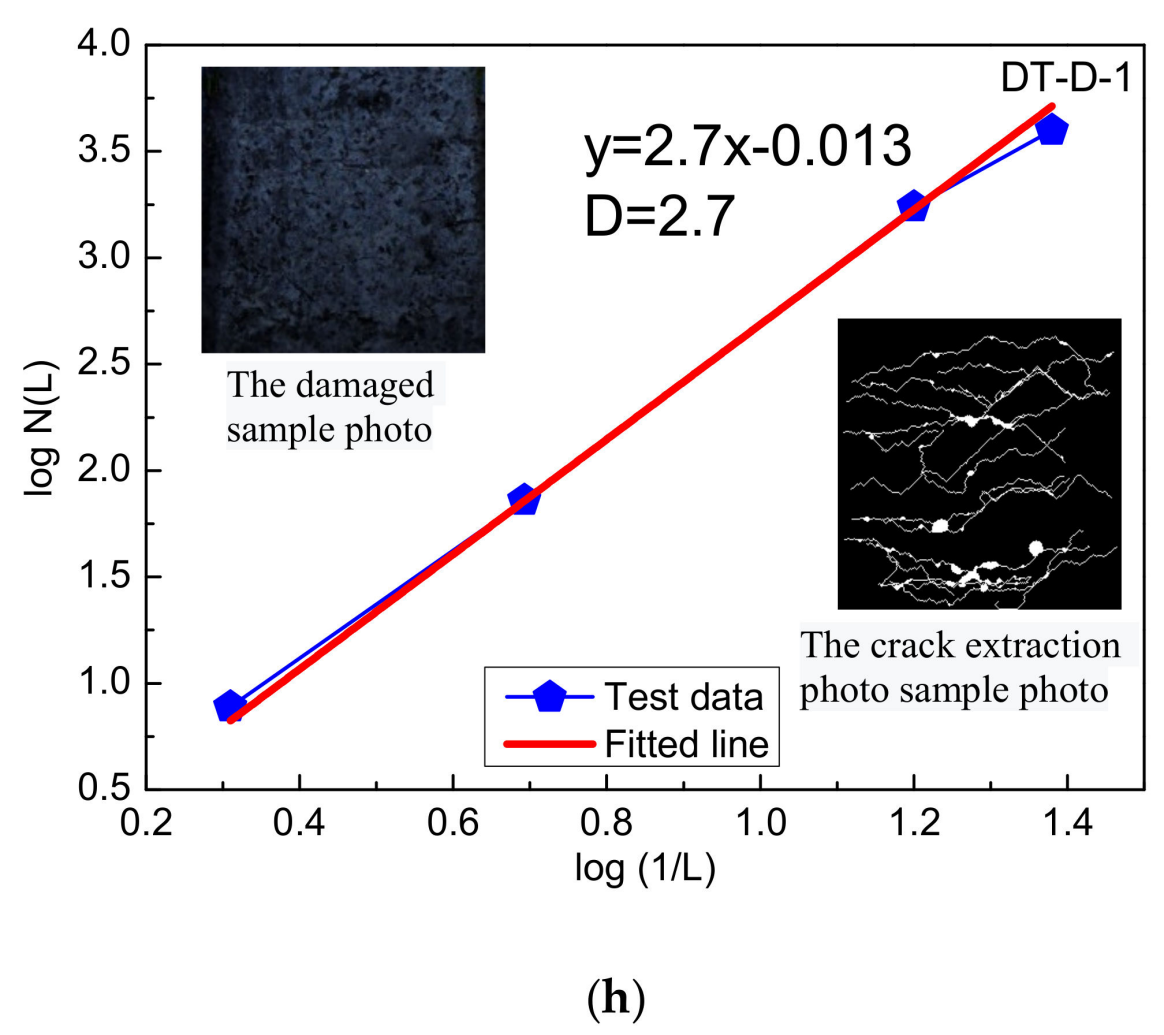
The fractal characteristics of rock damage at different loadings: (**a**,**c**,**e**,**g**) starting time of period-doubling bifurcation state of sample; (**b**,**d**,**f**,**h**) starting time of chaotic state of sample.

A FASTCAM SA-Z (200K-C-32 GB) high-speed camera system (Japan) was used to record the failure pattern of the samples during the loading process; photos are shown in Figure 9. Without obstructions on the sides of the samples in the SHPB apparatus, the failure mode of the half-circumference surface was randomly analyzed by the high-speed camera. To depict the development of rock cracks at different impact velocities, the high-speed camera system was used to photograph the sample failure process. Resultant images were processed by binary image processing technology [32], as shown in Figure 9.

The damage evolution process of rock sample is analyzed by the fractal geometry method. The fractal dimension values of the sample at the moment of period-doubling bifurcation and chaotic states are calculated, the crack binary images corresponding to the above moments are the measurement objects, with log (1/L) as the abscissa and log N(L) as the ordinate, and the log (1/L) and log N (L) curves are drawn, as shown in Figure 9a–h. As can be seen from the figure, there is a linear correlation between log (1/L) and log N(L), and the slope is the fractal dimension of each stress state.

#### 4.3.2. The Variation of Fractal Dimension

At different loading speeds, the sample damage evolution process has fractal characteristics and statistical self-similarity at the moment of period-doubling bifurcation and chaotic states because log (1/L) and log N (L) exhibit a linear correlation.

Rock materials have initial damage, and their damage fractal dimension is generally greater than 1. When the damage is evenly distributed absolutely, the fractal dimension is 3. When the damage distribution is more localized, the value is closer to 1; on the contrary, when the damage distribution is less localized, the value is closer to 3. Therefore, the fractal dimension of the damage reflects the degree of localization of the damage distribution.

**Figure 10:**
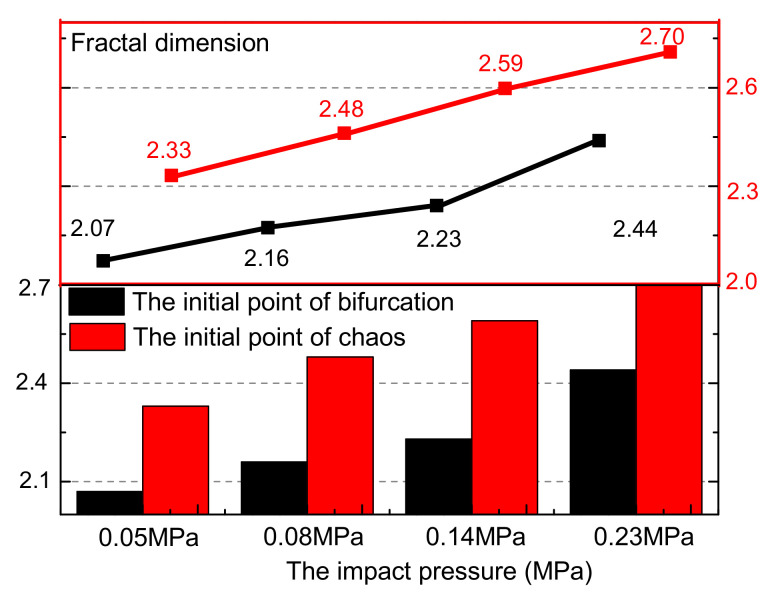
The fractal value at different loading conditions.

As shown in Figure 10, the damage fractal dimension value of the same sample is usually 0.26–0.36 times larger at the chaos initial moment than the initial moment of the period-doubling bifurcation, which indicates that the crack of the sample has a more discrete distribution during the whole loading process. The fractal dimension increases with the increase in impact pressure, from 2.07 at 0.05 MPa to 2.44 at 0.23 MPa, increasing by 17.87% at the start time of the period-doubling bifurcation state. The fractal dimension at the beginning of the chaotic state also shows the same increasing trend with the increase in impact pressure, which increases from 2.33 at 0.05 MPa to 2.70 at 0.23 MPa, and increases by 15.88%. This indicates that the increase in impact velocity obviously improves the homogenization degree of crack development in different stages of rock sample damage and failure process [33,34,35].

## 5. Conclusions

In this paper, the dynamic failure properties of the rock samples under different impact velocities were analyzed by the SHPB test system and sizing tests, based on chaos and fractal theory, the damage process and characteristics of the sample were analyzed, and the following conclusions were drawn:

1. Construction rock exhibits significant strain rate effects under dynamic loading conditions. As the impact pressure increases from 0.05 to 0.23 MPa, the peak stress of the sample increases from 82.19 to 284.16 MPa (an increase of 245.74%), and the increase process is in accordance with the power function law; the average particle size of the sample debris is reduced from 46.57 to 18.34 mm (reduced by 60.62%).
2. The damage–strain evolution information of the construction rock shows chaotic characteristics. The increase in the load velocity can delay the evolution of the generalized damage variable into the bifurcation state and the chaotic state.
3. The damage–strain evolution law (trend) of the sample from the initial moment of damage to the start of the period of bifurcation at different loading pressures shows similarity, and the slope of the fitted straight line is less than 7%.
4. Under dynamic loading conditions, the damage evolution process of the samples has fractal characteristics at the start of the bifurcation and chaotic periods, and the damage cracks have statistical self-similarity. Meanwhile, the increase in load velocity significantly increases the degree of homogenization of crack development in the process of damage.

## Figures and Tables

**Table 1:** The stress value of rock sample damage evolution.

Sample Number	Impact Pressure	Impact Velocity (m/s)	Peak Strength (MPa)	Stress Values Corresponding to Different Starting Times (MPa)		
	(MPa)			Damage	Period-Doubling Bifurcation	Chaotic
DT-A-1	0.05	3.52	81.10	36.1	71.2	79.4
DT-A-2	0.05	3.42	80.31	32.3	67.5	77.4
DT-A-3	0.05	3.46	85.17	37.6	73.3	82.3
DT-B-1	0.08	6.65	167.51	72.0	159.8	165.4
DT-B-2	0.08	6.91	159.91	78.3	145.7	155.7
DT-B-3	0.08	6.67	162.11	75.7	163.8	159.9
DT-C-1	0.14	9.71	237.02	119.4	231.7	236.3
DT-C-2	0.14	9.77	221.52	123.1	227.5	225.7
DT-C-3	0.14	9.90	230.33	117.9	237.9	227.8
DT-D-1	0.23	3.61	275.10	203.3	266.6	273.4
DT-D-2	0.23	3.67	287.38	207.9	273.7	284.9
DT-D-3	0.23	3.55	289.99	200.3	269.9	288.1

**Table 2:** The quality percentage of each grade of sample debris.

Sample Number	Impact Pressure (MPa)	m_vi_/%					m/g	Average Particle Diameter (mm)
		1	2	3	4	5		
DT-A-1	0.05	0.93%	1.31%	2.24%	4.29%	91.23%	536	46.48
DT-A-2	0.05	1.01%	1.11%	2.04%	4.63%	91.11%	540	46.45
DT-A-3	0.05	1.29%	1.10%	2.39%	6.99%	90.99%	544	46.77
DT-B-1	0.08	3.00%	2.81%	5.24%	16.67%	72.28%	534	39.16
DT-B-2	0.08	3.35%	2.97%	5.02%	15.43%	73.23%	538	39.45
DT-B-3	0.08	3.54%	3.54%	7.08%	15.27%	70.58%	537	38.27
DT-C-1	0.14	6.62%	6.17%	10.17%	24.36%	52.68%	539	31.04
DT-C-2	0.14	7.63%	5.97%	11.31%	25.08%	50.47%	543	30.13
DT-C-3	0.14	6.31%	6.77%	12.32%	20.61%	53.99%	536	31.31
DT-D-1	0.23	13.10%	10.41%	17.31%	31.65%	23.53%	532	18.31
DT-D-2	0.23	13.95%	10.52%	20.80%	31.07%	23.64%	535	18.55
DT-D-3	0.23	14.10%	11.81%	17.71%	34.04%	22.34%	543	18.16

## Data Availability

The introduction data supporting this manuscript are from previously reported studies and datasets, which have been cited. The processed data are available from the corresponding author upon request. The raw test data used to support the findings of this study are available from the corresponding author upon request.
